# Pulmonary tularemia with antineutrophil-cytoplasmic-antibody-negative pauci-immune crescentic glomerulonephritis causing acute kidney injury: a case report and review of the literature

**DOI:** 10.1186/s13256-025-05747-5

**Published:** 2025-12-17

**Authors:** Matthias Feyrer, Michael Städt, Pawlos Ichtiaris, Philipp Koehl, Alexander Schuh

**Affiliations:** 1 Department of Industrial Engineering and Health, Institute for Medical Engineering, OTH Technical University of Applied Sciences Amberg-Weiden, Hetzenrichter Weg 15, 92637 Weiden, Germany; 2 Department of Radiology, Neumarkt Hospital, 92318 Neumarkt, Germany; 3 Department of Radiology, Paracelsus Medical University Nuremberg, 90471 Nuremberg, Germany; 4 Nephrologic Center Marktredwitz and Selb, 95615 Marktredwitz, Germany; 5 Hospital of Trauma Surgery, Department of Orthopedics, Marktredwitz Hospital, 95615 Marktredwitz, Germany; 6 Hospital of Trauma Surgery, Department of Musculoskeletal Research, Marktredwitz Hospital, 95615 Marktredwitz, Germany

**Keywords:** Tularemia, *Francisella tularensis*, ANCA-negative pauci-immune crescentic glomerulonephritis, Acute kidney injury, Case report

## Abstract

**Background:**

Tularemia is a zoonotic disease that is rarely diagnosed in Europe. Its clinical presentation is highly variable, requiring consideration of a broad differential diagnosis.

**Methods:**

We present a case report of a pulmonary tularemia with antineutrophil-cytoplasmic-antibody-negative pauci-immune crescentic glomerulonephritis causing acute kidney injury, and a systematic review of the literature.

**Case report:**

We report the case of a 73-year-old white man who presented with pneumonia, sepsis and acute kidney injury due to a pauci-immune crescentic glomerulonephritis with negativity for antineutrophil cytoplasmic antibodies. Initial management with dialysis, empirical antibiotics, and immunosuppression was adjusted after identification of *Francisella tularensis*, and targeted antibiotic therapy was administered successfully. At 4 years of follow-up, no recurrence was observed.

**Literature review:**

Our review of the literature identified only a few case reports of tularemia complicated by acute kidney injury. None documented biopsy-proven pauci-immune crescentic glomerulonephritis.

**Conclusion:**

Pulmonary tularemia complicated by acute kidney injury is an uncommon clinical constellation. Biopsy-proven pauci-immune crescentic glomerulonephritis with antineutrophil-cytoplasmic-antibody-negativity in this setting appears to be very rare. Careful differential diagnosis, early recognition of tularemia, and timely initiation of effective antibiotic therapy are critical to achieving favorable outcomes.

**Supplementary Information:**

The online version contains supplementary material available at 10.1186/s13256-025-05747-5.

## Background

Tularemia is a zoonotic disease caused by *Francisella tularensis* (*F. tularensis*), a Gram-negative rod-shaped or coccoid bacterium [1–3]. Its incidence in Europe, including Germany, is very low [4–6]. In addition, diagnosis is often challenging owing to the nonspecific clinical presentation [7].

Renal involvement in tularemia has been reported only rarely, with a few patient-level cases of acute kidney injury (AKI) documented, most often in the context of sepsis, multiorgan dysfunction, or rhabdomyolysis [8–14]. To our knowledge, biopsy-proven pauci-immune crescentic glomerulonephritis in the context of tularemia has not been previously reported. We therefore present this constellation together with a systematic review of the literature.

## Methods

We report the case of a 73-year-old man from Bavaria (Germany) with tularemia who presented with pneumonia, sepsis, and an episode of acute renal failure in the form of a pauci-immune crescentic glomerulonephritis with negativity for antineutrophil cytoplasmic antibodies (ANCA).

We also performed a systematic literature search in PubMed, MEDLINE (via EBSCO), and Scopus to identify case reports of tularemia with renal involvement. Search strategies combined the terms for tularemia/*Francisella tularensis* with renal outcomes (acute kidney injury, renal failure, glomerulonephritis, crescentic glomerulonephritis, vasculitis, ANCA, and pauci-immune) and excluded experimental/animal studies, reports without renal involvement, and studies on transplant or graft recipients. No restrictions regarding publication date or language were applied. The last search was conducted on 23 September 2025. The complete Boolean search string for PubMed is shown in Table 1 as an example, while corresponding search strategies for MEDLINE and Scopus are provided in the Supplementary Material.

**Table 1:** Complete Boolean search strategy for PubMed used in the systematic literature review (final search date: 23 September 2025; corresponding full search strategies for MEDLINE and Scopus are provided in the Supplementary Material)

Boolean operator	Search terms
	(“Tularemia”[MeSH Terms] OR “*Francisella tularensis*”[MeSH Terms] OR “tularemia”[Title/Abstract] OR “tularaemia”[Title/Abstract] OR “*Francisella tularensis*”[Title/Abstract])
AND	(“Acute Kidney Injury”[MeSH Terms] OR “Renal Insufficiency”[MeSH Terms] OR “Glomerulonephritis”[MeSH Terms] OR “Vasculitis”[MeSH Terms] OR “Antibodies, Antineutrophil Cytoplasmic”[MeSH Terms] OR “acute kidney injury”[Title/Abstract] OR “AKI”[Title/Abstract] OR “renal failure”[Title/Abstract] OR “kidney failure”[Title/Abstract] OR “glomerulonephritis”[Title/Abstract] OR (“crescent*”[Title/Abstract] AND “glomerulonephr*”[Title/Abstract]) OR “vasculitis”[Title/Abstract] OR “ANCA”[Title/Abstract] OR “anti-neutrophil cytoplasmic”[Title/Abstract] OR “pauci-immune”[Title/Abstract] OR “pauciimmune”[Title/Abstract])
NOT	(“transplant*”[Title/Abstract] OR “graft*”[Title/Abstract])
AND	“humans”[MeSH Terms]

**Fig. 1:**
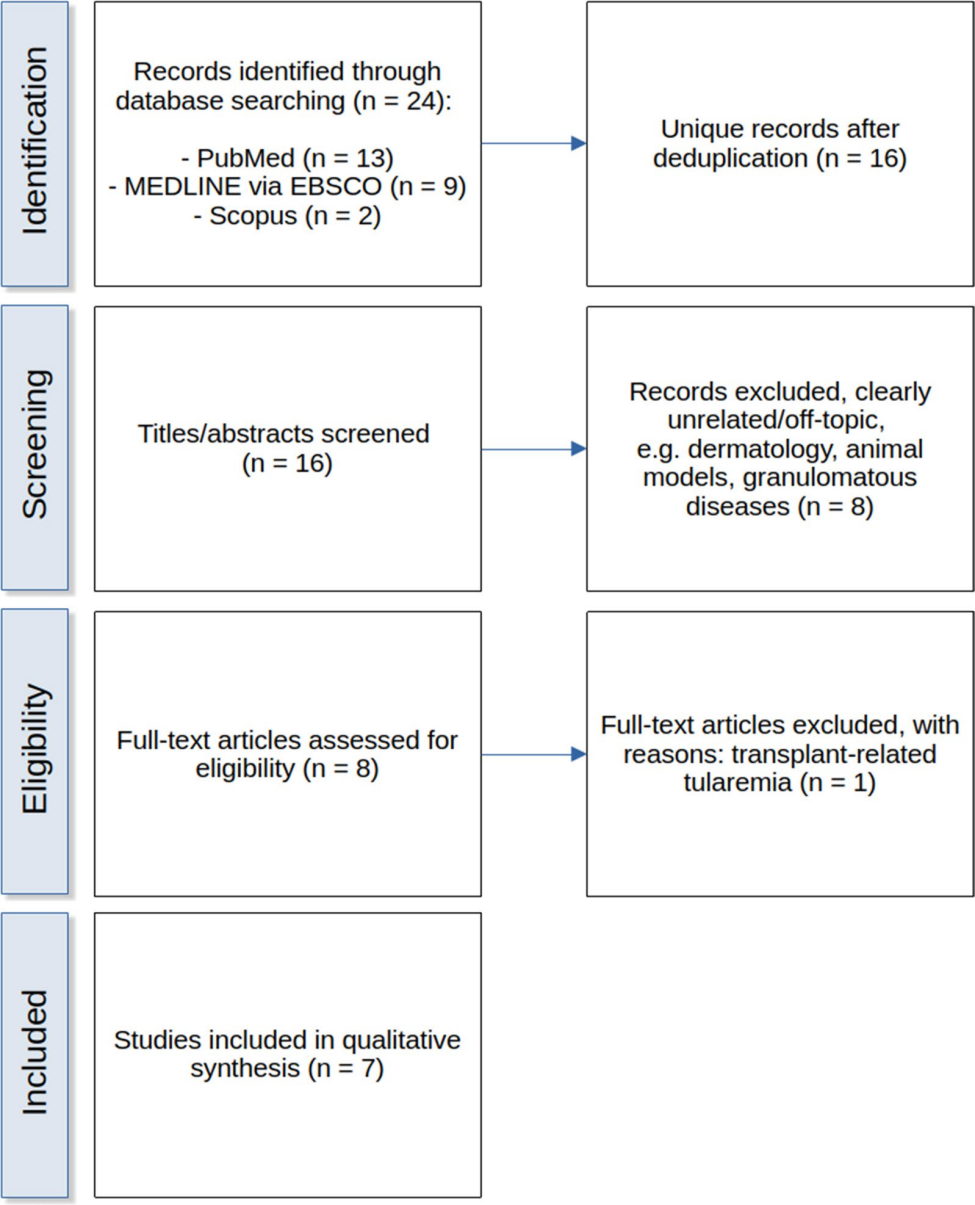
Preferred Reporting Items for Systematic reviews and Meta-Analyses flow diagram of the literature search and study selection process

The search yielded 13 records in PubMed, 9 in MEDLINE, and 2 in Scopus. After deduplication, 16 unique records remained. Titles and abstracts were screened, and eight articles were selected for full-text review. Of these, seven case reports/series describing human tularemia with AKI were included, while one report was excluded after full-text review owing to transplant-related immunosuppression. The study selection process is illustrated in a Preferred Reporting Items for Systematic reviews and Meta-Analyses (PRISMA) flow diagram (Fig. 1).

## Case report

A 73-year-old white male patient was admitted to our emergency department in early March 2021 complaining of a 5-day history of chills, productive cough with yellow sputum, shortness of breath at rest, and lower limb pain. The patient had no known relevant preexisting medical conditions and neither the psychosocial nor the family history revealed any notable findings. Physical examination revealed fever, purulent conjunctivitis of the right eye, and a fresh but non-inflamed wound on the distal left thigh sustained during forestry work approximately 10 days prior to admission. The patient also reported a possible recent tick bite, but denied contact with hares or other wild animals.

**Fig. 2:**
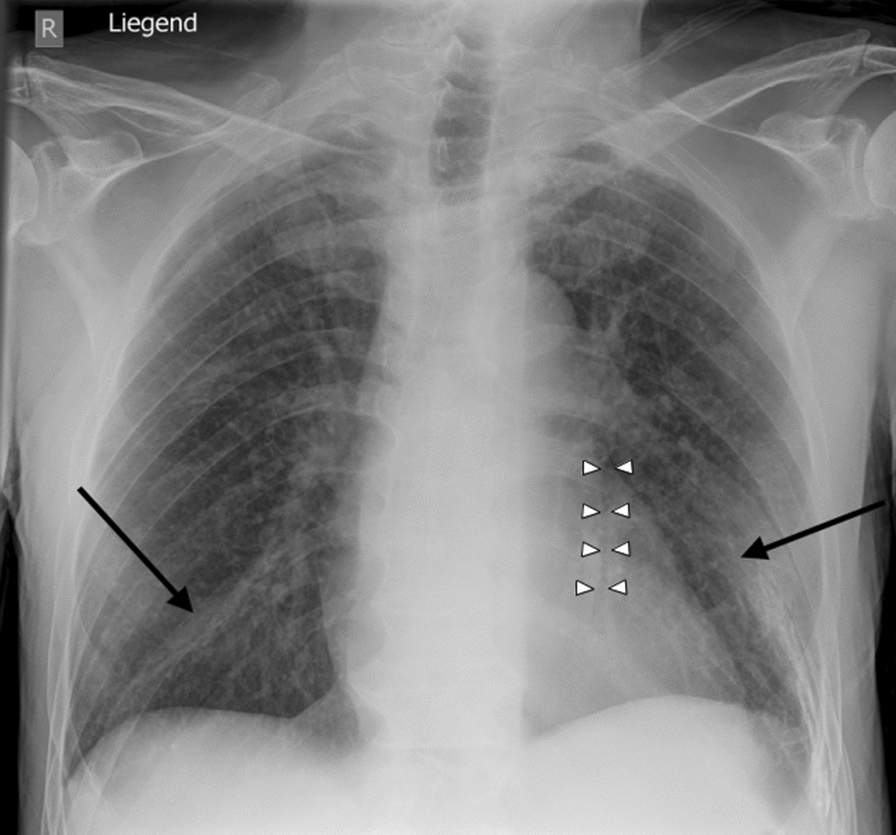
Chest X-ray (supine) from day 1 showing only isolated areas of decreased transparency (black arrows) and a positive retrocardial air bronchogram (white arrows)

**Fig. 3:**
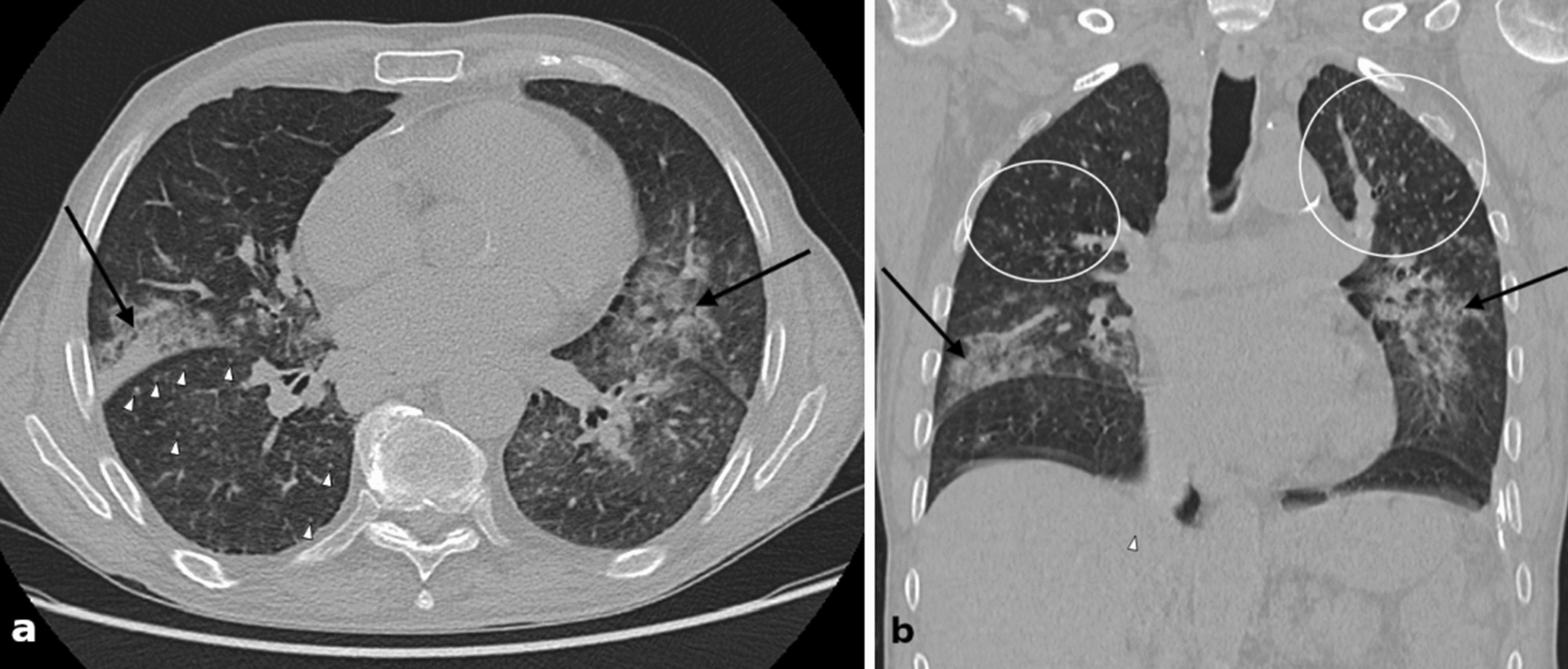
Chest computed tomography (axial [**a**] and coronal [**b**] non-contrast enhanced source images) from day 2 showing bilateral patchy consolidations (black arrows) and subtle micronodules, which are difficult to distinguish from small pulmonary vessels (white arrows and white ovals)

Initial laboratory tests showed impaired renal function and elevated inflammatory markers (creatinine 6.77 mg/dl corresponding estimated glomerular filtration rate [eGFR] 8 ml/minute/1.73 m^2^, urea 180 mg/dl, C-reactive protein [CRP] 306 mg/l), with a normal leukocyte count (8.8 × 10^3^/μl). Initial infectious work-up showed no evidence of coronavirus disease 2019 (COVID-19) or other acute infections. Urinalysis revealed proteinuria and hematuria; urine sediment contained a few eumorphic red blood cells and crystals. Quantitative proteinuria (24-hour collection or spot protein/creatinine ratio) was not available. Abdominal ultrasound revealed bilaterally enlarged kidneys with mildly increased parenchymal echogenicity, consistent with acute renal failure. Emergency dialysis treatment was initiated via a Shaldon catheter. Electrocardiogram, cardiac and venous ultrasound were unremarkable, as was a subsequent transesophageal echocardiogram to rule out endocarditis. Chest X-ray showed isolated, nonspecific opacities, interpreted as infiltrates, left-sided accompanied by a matching positive retrocardial air bronchogram (Fig. 2). Empirical antibiotic therapy with piperacillin/tazobactam and clarithromycin was initiated for suspected atypical pneumonia, but clinical response was minimal. Non-contrast enhanced chest computed tomography (CT) in 2.5 mm slices revealed bilateral consolidations and infiltrates, along with subtle micronodules, which were difficult to distinguish from small lung vessels (Fig. 3). Additional tests revealed antineutrophil cytoplasmatic antibodies by indirect immunofluorescence (IIF) with fine-speckled nuclear pattern with positive chromatin region, in the form of an initially low-titer pANCA of 1:32, rising to 1:320 on repeat testing, while myeloperoxidase (MPO-) and proteinase 3 (PR3)-ANCA remained consistently negative by enzyme-linked immunosorbent assay (ELISA). Anti-GBM antibodies were negative. Complement analysis was performed once during the acute phase (day 7) and showed normal C3 and C4 values; no convalescent samples were obtained. Cryoglobulins were normal, soluble interleukin-2 (IL-2) receptor was elevated, and angiotensin-converting enzyme (ACE) was within the normal range.

A renal biopsy comprising two cores with 14 glomeruli was obtained. One glomerulus was globally sclerotic, while 4/13 showed cellular crescents with fibrinoid necrosis, consistent with focal–segmental, extracapillary proliferative and necrotizing pauci-immune glomerulonephritis. The remaining glomeruli appeared preserved. Immunofluorescence revealed no significant deposits of IgG, IgA, IgM, C3c, or C1q, apart from minimal mesangial IgM reactivity. No evidence of immune-complex or complement-mediated glomerulonephritis, amyloidosis, or light-chain–related disease was found. Approximately 10% interstitial fibrosis/tubular atrophy and mild arteriosclerosis were noted. Electron microscopy was not performed owing to the clear light- and immunohistological findings; however, the latter images were not available for publication despite attempts to retrieve them from the original pathology archive. Overall, the biopsy showed pauci-immune crescentic glomerulonephritis. Given persistently negative MPO- and PR3-ANCA, this was classified as ANCA-negative. At this stage, treatment with intravenous corticosteroids was initiated together with a single session of plasmapheresis, while pulse cyclophosphamide was scheduled.

However, directly after this plasmaperhesis, results from blood cultures positive for *F. tularensis* arrived on the same day. In parallel, serological testing during the second week after admission demonstrated IgM and IgG positivity, consistent with acute tularemia. Additional serological testing by enzyme immunoassay (EIA) repeatedly revealed isolated Puumala virus IgM positivity without seroconversion to IgG antibodies, which the diagnostic laboratory interpreted as a nonspecific finding. In light of these findings, corticosteroids were tapered and discontinued, and the immunosuppressive regimen was curtailed before cyclophosphamide would be administered. Instead, antibiotic therapy was switched to intravenous ciprofloxacin (plus ceftriaxone), which could be later transitioned to oral administration and led to a marked improvement in both inflammatory markers and renal function. Final confirmation was subsequently obtained from the German National Reference Laboratory for Tularemia (Robert Koch Institute, Berlin), including real-time polymerase chain reaction (PCR) for the *F. tularensis* markers fopA and tul4 and region of interest 1 (RD1)-PCR for subspecies differentiation (*F. tularensis* subsp. *holarctica*). Susceptibility testing was performed with a non-accredited method and interpreted according to the Clinical and Laboratory Standards Institute (CLSI) criteria, demonstrating sensitivity to aminoglycosides, fluoroquinolones, doxycycline, chloramphenicol, and streptomycin. Intermittent hemodialysis was required for a total of seven sessions (on days 1–3, 5, 7, 9, and 11), initially via a Shaldon catheter and later via a tunneled atrial catheter. Thereafter, creatinine stabilized at around 2.70 mg/dl (corresponding eGFR 24 ml/minute/1.73 m^2^), and with normalization of urine output, dialysis could be discontinued after a trial withdrawal, followed by catheter removal. Given the biopsy-proven rapidly progressive glomerulonephritis, interpreted as an immunological manifestation in the context of tularemia-associated sepsis, low-dose oral corticosteroids were reintroduced to support renal recovery. Both the thigh wound and the conjunctivitis remained uncomplicated during hospitalization and improved with local supportive measures. As clinical and laboratory parameters continued to improve, the patient was discharged home on day 24 of hospitalization. Corticosteroids were successfully tapered over the following months, ultimately resulting in full recovery. At 4-year follow-up, the patient remains asymptomatic, with normalized inflammatory markers and renal function.

The clinical timeline is summarized in Table 2, detailing the sequence of findings, treatments, and outcomes.

**Table 2:** Structured clinical timeline summarizing symptoms, diagnostics, interventions, and outcomes during hospitalization and follow-up

Day	Event
~Day −5	*Onset of symptoms*: chills, productive cough with yellow sputum, shortness of breath, lower limb pain
Day 1	Admission *Clinical findings*: fever, cough, shortness of breath, right-sided conjunctivitis, left-sided thigh wound *Laboratory findings*: AKI (creatinine 6.77 mg/dl corresponding eGFR 8 ml/minute/1.73 m^2^, CRP 306 mg/l) with anuria, proteinuria, hematuria *Imaging*: ultrasound with enlarged kidneys, X-ray with bilateral infiltrates *Therapy*: emergency Shaldon catheter, initiation of intermittent hemodialysis; empirical antibiotics (piperacillin/tazobactam + clarithromycin) for suspected atypical pneumonia
Day 2	*Chest CT*: bilateral consolidations, infiltrates and subtle micronodules
Day 9	*Results from kidney biopsy*: pauci-immune focal–segmental crescentic glomerulonephritis
Day 10	*Therapy*: initiation of intravenous corticosteroids and plasmapheresis, directly afterward: *Results from blood culture*: positive for *F. tularensis*, sensitive for fluoroquinolones among others (later confirmed by the German National Reference Laboratory for Tularemia, Robert Koch Institute, Berlin, for *F. tularensis* subsp. *holarctica*) *Therapy*: antibiotics switched to intravenous ciprofloxacin (+ ceftriaxone) Cyclophosphamide withheld, plasmapheresis discontinued
Day 11	*Therapy*: insertion of tunneled atrial dialysis catheter
Day 12–14	*Clinical course*: stabilization of creatinine (2.70 mg/dl corresponding eGFR 24 ml/minute/1.73 m^2^), progressive improvement of urine output to normal levels, dialysis trial withdrawal
Day 14	*Result from serology*: *F. tularensis* IgM and IgG positive
Day 15–22	*Clinical course*: further improvement of clinical and laboratory parameters *Therapy*: corticosteroids reintroduced at low dose to support recovery in the context of biopsy-proven crescentic glomerulonephritis
Day 23	*Therapy*: removal of tunneled atrial dialysis catheter Completion of 14-day course of ciprofloxacin (+ ceftriaxone)
Day 24	*Discharge*: in stable condition (creatinine 1.69 mg/dl corresponding eGFR 42 ml/minute/1.73 m^2^, CRP 37 mg/l)
~Month 5	*Clinical course*: corticosteroids fully tapered. Clinical remission achieved
Year 1, 2, 3, 4	*Long-term follow-up*: complete recovery, no relapse, normal renal and inflammatory markers

## Literature review

Our systematic review identified seven relevant reports of tularemia with AKI: Tilley *et al*. (1983) described survival of a patient with AKI with clinically suspected, but not histologically confirmed, interstitial nephritis [8]. The Clinicopathologic Conference from Barnes Hospital (1984) reported a fatal case of typhoidal tularemia with dialysis-dependent AKI; renal histology from autopsy was not fully detailed, but renal cortical necrosis was considered more likely than acute tubular necrosis [10]. Kaiser *et al*. (1985) presented four cases of rhabdomyolysis-associated renal failure with variable therapy and outcomes [9]. Georgievskii *et al*. (1988) documented survival in a patient with renal involvement of unclear type managed by hemodialysis without antibiotics owing to late diagnosis by seroconversion [11]. Lee *et al*. (1991) reported pulmonary tularemia complicated by AKI successfully treated with imipenem/cilastatin [12]. Onuigbo *et al*. (2002) described fatal sepsis with biopsy-proven acute tubular necrosis (ANCA-negative) [13]. Ørbæk *et al*. (2020) presented two cases, one without renal involvement and one with secondary AKI due to myocarditis and pulmonary edema without need for dialysis [14]. None of these cases documented biopsy-proven pauci-immune crescentic glomerulonephritis. Further details are summarized in Table 3.

**Table 3:** Published case reports and case series of tularemia with AKI or GN, together with the present case (references as cited in the text)

Author	Year of disease	Country	Age/sex	Tularemia manifestation	Renal involvement	Renal histology	Antibiotics	Outcome
Tilley *et al*. [8]	1980	USA	71 M	Pulmonary	Dialysis-near non-oliguric AKI	None acquired, IN clinically suggested	Tobramycin, ampicillin → doxycycline, (erythromycin)	Survival
CPC Barnes (1984)	1983	USA	65 M	Typhoidal with sepsis	Dialysis-dependent AKI (peritoneal dialysis), additional rhabdomyolysis	Autopsy: renal histology not exactly reported, RCN versus ATN discussed	Cefamandole → piperacillin, oxacillin, tobramycin	Fatal
Kaiser *et al*. [9]	1978–1982 (four cases)	USA	1: 58 M 2: 63 F 3: 49 M 4: 31 M	1: Pulmonary/ulceroglandular 2: Typhoidal with sepsis 3–4: Pulmonary	All: AKI due to rhabdomyolysis; 2: dialysis-dependent	2: Autopsy, no renal histology reported; others: none acquired	1: Penicillin G, oxacillin, gentamicin 2: Cefazolin, tobramycin → chloramphenicol, cefotaxime 3: Cefamandole → tobramycin 4: Penicillin G, tobramycin	2: Fatal; others: survival
Georgievskii *et al*. [11]	1986	USSR	25 M	Pulmonary	Dialysis-dependent AKI	None acquired	None (only supportive therapy owing to late diagnosis by seroconversion)	Survival
Lee *et al*. [12]	1990	USA	57 M	Pulmonary	AKI without dialysis-dependency	None acquired	Cefazolin, imipenem/cilastatin → erythromycin, vancomycin → imipenem/cilastatin	Survival (normal 1-year follow-up)
Onuigbo *et al*. [13]	2002	USA	46 M	Pulmonary with sepsis	Dialysis-dependent AKI	ATN (no GN, ANCA negative)	Doxycycline, levofloxacin → + gentamicin	Fatal
Ørbæk *et al*. [14]	2019 (two cases)	Denmark	1: 63 F 2: 76 F	1: Ulceroglandular 2: Typhoidal	1: None 2: Secondary AKI due to myocarditis, heart failure, pulmonary edema; no dialysis-dependency	None acquired	1: Doxycycline 2: Piperacillin/tazobactam, amoxicillin/clavulanic acid, doxycycline	1–2: Survival, (almost normal 3-month follow-up)
Present case	2021	Germany	73 M	Pulmonary with sepsis	Dialysis-dependent AKI	Pauci-immune crescentic GN	Ciprofloxacin and ceftriaxone, corticosteroids	Survival, (normal 4-year follow-up)

AKI, acute kidney injury; ATN, acute tubular necrosis; CPC, clinicopathologic conference; GN, glomerulonephritis; IN, interstitial nephritis; RCN, renal cortical necrosis

## Discussion

In our case, the initial working diagnosis of atypical pneumonia was revised to pauci-immune, ANCA-negative crescentic glomerulonephritis, primarily on the basis of the results of the kidney biopsy. At that stage, in the absence of an identified infectious trigger, the clinical presentation was indistinguishable from a primary small-vessel vasculitis, and high-dose corticosteroids with cyclophosphamide were considered the standard of care. Cyclophosphamide induction therapy was therefore planned but ultimately withheld after subsequent blood cultures confirmed *F. tularensis* subsp. *holarctica*. This diagnostic clarification was critical, as the patient could instead be managed successfully with targeted antibiotic therapy [15–17].

Renal involvement in tularemia is considered rare. It remains unclear whether *F. tularensis* may have triggered the pauci-immune glomerulonephritis. Studies from the COVID-19 pandemic describe vasculitides of varying pathogenesis after viral infection in the form of pauci-immune crescentic glomerulonephritis and rarely after mRNA vaccination [18, 19]. These findings suggest that immunological triggers may not be limited to viral infections, but may also arise in bacterial or other infectious contexts. In bacterial infections, proposed mechanisms include molecular mimicry and immune activation via bacterial superantigens [20]. Such links have been described for *Staphylococcus aureus*, where toxic shock syndrome toxin-1 (TSST-1)-producing strains increase relapse risk in granulomatosis with polyangiitis [21], and for *Bartonella henselae* endocarditis, which has been associated with PR3-ANCA positivity and crescentic glomerulonephritis [22]. However, in our patient, both PR3- and MPO-ANCA remained negative, raising the possibility of transient, unspecific ANCA reactivity or molecular mimicry phenomena such as those described for lysosome-associated membrane protein-2 (LAMP-2) in pauci-immune necrotizing glomerulonephritis [23]. Alternatively, sepsis itself—independent of ANCA specificity—may act as a potential trigger for crescentic glomerulonephritis [24, 25]. From a Bradford–Hill perspective, temporality and plausibility support an association, but strength and specificity of evidence are insufficient to infer causality.

The most likely route of infection in our patient was direct inoculation at the site of a forestry-related thigh wound or by a recent tick bite, in line with the epidemiology of tularemia in Central Europe, where transmission occurs via contact with contaminated animal tissue or arthropod vectors (ticks, deer flies) [26]. Given the dominant pulmonary manifestation, we framed our case as pulmonary tularemia. However, in light of the possible entry routes and the presence of sepsis, an alternative classification as typhoidal tularemia could also be considered. In addition, the patient presented with a right-sided conjunctivitis, a finding consistent with ocular involvement as seen in oculoglandular tularemia [3, 27].

Only qualitative proteinuria data were accessible, as no 24-hour measurement or spot protein/creatinine ratio had been documented. Likewise, laboratory investigations were partly performed by an external reference laboratory using standard diagnostic IIF and ELISA methods, with ANCA titers < 1:10 considered negative. Detailed assay specifications and manufacturer information were not available from archived records. Notably, isolated Puumala virus IgM positivity was detected but did not seroconvert. According to the interpreting laboratory, this pattern was considered most consistent with an unspecific or cross-reactive finding rather than evidence of acute hantavirus infection. These methodological constraints limit fine-grained interpretation of serologic and urinary findings.

The differential diagnosis was further complicated by overlapping renal and pulmonary findings. Imaging features of tularemia pneumonia can closely mimic tuberculosis, sarcoidosis, pulmonary malignancy, or nonspecific findings [28–30]. This underscores the importance of considering tularemia in endemic settings or after relevant exposure, even when the clinical and radiological picture is atypical or nonspecific.

Antibiotic therapy remains the cornerstone of tularemia management, with fluoroquinolones regarded as first-line agents and aminoglycosides or tetracyclines as alternatives [15, 16, 31, 32]. In our patient, a 14-day-course with ciprofloxacin was initiated (in line with German recommendations [17]) and combined with ceftriaxone to provide broad-spectrum coverage in the context of sepsis. Aminoglycosides were avoided owing to concomitant AKI, and tetracyclines were considered less suitable given the severity of disease. The favorable course highlights the importance of timely initiation of an active agent.

Renal involvement has been reported only sporadically in literature, as our systematic literature review of case reports and small case series shows. Historical epidemiological series similarly reported renal involvement only sporadically [33, 34]; a finding corroborated by a recent retrospective study covering tularemia cases from 1993 to 2023 [35]. Most previously published case reports describe AKI in the setting of sepsis, multiorgan dysfunction, or rhabdomyolysis, and only in isolated cases was histology available. Compared with previously published cases, which often lacked histological confirmation, our report is distinguished by biopsy-proven pauci-immune crescentic glomerulonephritis.

## Conclusion

Pulmonary tularemia complicated by AKI is an uncommon clinical constellation. Biopsy-proven pauci-immune crescentic glomerulonephritis with ANCA-negativity in this setting appears to be very rare. Careful differential diagnosis, early recognition of tularemia, and timely initiation of effective antibiotic therapy are critical to achieving favorable outcomes.

**Table:** 

What this case adds	
• *Pitfalls of relying on low-titer pANCA* In the setting of persistently negative MPO- and PR3-ANCA and systemic infection, a low-titer pANCA should not be over-interpreted as evidence of primary ANCA-associated vasculitis • *Chest CT mimics in pulmonary tularemia*: pulmonary tularemia can radiologically resemble tuberculosis, sarcoidosis, malignancy, or present with entirely nonspecific findings, complicating differential diagnosis • *When to de-escalate immunosuppression*: in suspected or confirmed infection, early reconsideration and timely withdrawal of immunosuppressive therapy are critical to avoid unnecessary toxicity • *Timely pathogen identification changes management*: early detection of *F. tularensis* enables targeted antibiotic therapy and is decisive for a favorable outcome	

## Supplementary Information

Additional file 1.

Additional file 2.

## Data Availability

The data/material supporting the findings of this case report are available from the corresponding author upon reasonable request. The complete search strategies are provided in the Supplementary Material.
