# Supplementary material for: Pulmonary tularemia with antineutrophil-cytoplasmic-antibody-negative pauci-immune crescentic glomerulonephritis causing acute kidney injury: a case report and review of the literature
Source: J Med Case Rep. 2025 Dec 17;20:38. doi: 10.1186/s13256-025-05747-5 (PMC12822207; doi:10.1186/s13256-025-05747-5)
Supplement: Supplementary file 1 — Additional file 1. [file 13256_2025_5747_MOESM1_ESM.docx]

**Supplementary material**

### Search strategy

The following Boolean search strings were used to identify reports of tularemia with renal involvement. Searches were conducted in PubMed, MEDLINE (via EBSCO), and Scopus, with no restrictions on date or language. The last search was performed on 23 September 2025.

PubMed:

( "Tularemia"[MeSH Terms] OR "Francisella tularensis"[MeSH Terms] OR "tularemia"[Title/Abstract] OR "tularaemia"[Title/Abstract] OR "Francisella tularensis"[Title/Abstract] )

AND ( "Acute Kidney Injury"[MeSH Terms] OR "Renal Insufficiency"[MeSH Terms] OR "Glomerulonephritis"[MeSH Terms] OR "Vasculitis"[MeSH Terms] OR "Antibodies, Antineutrophil Cytoplasmic"[MeSH Terms] OR "acute kidney injury"[Title/Abstract] OR "AKI"[Title/Abstract] OR "renal failure"[Title/Abstract] OR "kidney failure"[Title/Abstract] OR "glomerulonephritis"[Title/Abstract] OR ("crescent*"[Title/Abstract] AND "glomerulonephr*"[Title/Abstract]) OR "vasculitis"[Title/Abstract] OR "ANCA"[Title/Abstract] OR "anti-neutrophil cytoplasmic"[Title/Abstract] OR "pauci-immune"[Title/Abstract] OR "pauciimmune"[Title/Abstract] )

NOT ( "transplant*"[Title/Abstract] OR "graft*"[Title/Abstract] ) AND "humans"[MeSH Terms]

MEDLINE (via EBSCO):

( MH "Tularemia" OR MH "Francisella tularensis"

OR TI tularemia OR AB tularemia

OR TI tularaemia OR AB tularaemia

OR TI "Francisella tularensis" OR AB "Francisella tularensis" )

AND ( MH "Acute Kidney Injury" OR MH "Renal Insufficiency"

OR MH "Glomerulonephritis" OR MH "Vasculitis"

OR MH "Antibodies, Antineutrophil Cytoplasmic"

OR TI "acute kidney injury" OR AB "acute kidney injury"

OR TI AKI OR AB AKI

OR TI "renal failure" OR AB "renal failure"

OR TI "kidney failure" OR AB "kidney failure"

OR TI glomerulonephritis OR AB glomerulonephritis

OR (TI crescent* AND TI glomerulonephr*)

OR (AB crescent* AND AB glomerulonephr*) )

NOT ( TI transplant* OR AB transplant* OR TI graft* OR AB graft* )

Scopus:

TITLE-ABS-KEY ( tularemia OR tularaemia OR "Francisella tularensis" OR Francisella )

AND TITLE-ABS-KEY ( "acute kidney injury" OR AKI OR "renal failure" OR "kidney failure" OR glomerulonephritis OR "crescentic glomerulonephritis" OR vasculitis OR ANCA OR "anti-neutrophil cytoplasmic" OR "pauci-immune" OR "pauci immune" OR pauciimmune ) AND TITLE-ABS-KEY ( human )

NOT TITLE-ABS-KEY ( transplant* OR graft* )
