# Supplementary material for: Pulmonary tularemia with antineutrophil-cytoplasmic-antibody-negative pauci-immune crescentic glomerulonephritis causing acute kidney injury: a case report and review of the literature
Source: J Med Case Rep. 2025 Dec 17;20:38. doi: 10.1186/s13256-025-05747-5 (PMC12822207; doi:10.1186/s13256-025-05747-5)
Supplement: Supplementary file 2 — Additional file 2. [file 13256_2025_5747_MOESM2_ESM.pdf]

CARE Checklist of information to include when writing a case report

CARE case report guidelines

(cc) BY-NC-ND

Topic	Item	Checklist item description	Reported on Line
Title	1	The diagnosis or intervention of primary focus followed by the words "case report"	1-2
Key Words	2	2 to 5 key words that identify diagnoses or interventions in this case report, including "case report"	47-48
Abstract (no references)	3a	Introduction: What is unique about this case and what does it add to the scientific literature?	30-31
	3b	Main symptoms and/or important clinical findings	33-34
	3c	The main diagnoses, therapeutic interventions, and outcomes	35-37
	3d	Conclusion—What is the main "take-away" lesson(s) from this case?	41-45
Introduction	4	One or two paragraphs summarizing why this case is unique (**may include references**)	50-62
Patient Information	5a	De-identified patient specific information.	65
	5b	Primary concerns and symptoms of the patient.	66-67
	5c	Medical, family, and psycho-social history including relevant genetic information	67-68
	5d	Relevant past interventions with outcomes	67-68
Clinical Findings	6	Describe significant physical examination (PE) and important clinical findings.	68-70
Timeline	7	Historical and current information from this episode of care organized as a timeline	65-70
Diagnostic Assessment	8a	Diagnostic testing (such as PE, laboratory testing, imaging, surveys).	71-91
	8b	Diagnostic challenges (such as access to testing, financial, or cultural)	92-93
	8c	Diagnosis (including other diagnoses considered)	94-95
	8d	Prognosis (such as staging in oncology) where applicable	95-97
Therapeutic Intervention	9a	Types of therapeutic intervention (such as pharmacologic, surgical, preventive, self-care)	95-97
	9b	Administration of therapeutic intervention (such as dosage, strength, duration)	95-97
	9c	Changes in therapeutic intervention (with rationale)	100-103
Follow-up and Outcomes	10a	Clinician and patient-assessed outcomes (if available)	104-107
	10b	Important follow-up diagnostic and other test results	107-108
	10c	Intervention adherence and tolerability (How was this assessed?)	107-108
	10d	Adverse and unanticipated events	107-108
Discussion	11a	A scientific discussion of the strengths AND limitations associated with this case report	166-171
	11b	Discussion of the relevant medical literature **with references**.	185-195
	11c	The scientific rationale for any conclusions (including assessment of possible causes)	198-201
	11d	The primary "take-away" lessons of this case report (without references) in a one paragraph conclusion	198-201
Patient Perspective	12	The patient should share their perspective in one to two paragraphs on the treatment(s) they received	107-108
Informed Consent	13	Did the patient give informed consent? Please provide if requested	Yes ☒ No ☐
